# Gradual Recovery from Nonambulatory Quadriparesis Caused by Metastatic Epidural Cervical Cord Compression in an Octogenarian Gallbladder Carcinoma Patient Treated with Image-Guided Three-Dimensional Conformal Radiotherapy Alone Using a Field-in-Field Technique

**DOI:** 10.1155/2014/398208

**Published:** 2014-08-11

**Authors:** Kazuhiro Ohtakara, Hiroaki Hoshi

**Affiliations:** ^1^Department of Radiation Oncology, Murakami Memorial Hospital, Asahi University, 3-23 Hashimoto-cho, Gifu 500-8523, Japan; ^2^Department of Radiation Oncology, Chuno Kosei Hospital, 5-1 Wakakusadori, Seki 501-3802, Japan; ^3^Department of Radiology, Gifu University Graduate School of Medicine, 1-1 Yanagido, Gifu 501-1194, Japan

## Abstract

Radiotherapy for acute metastatic epidural spinal cord compression (MESCC) involves conventional techniques and dose fractionation schemes, as it needs to be initiated quickly. However, even with rapid intervention, few paraplegic patients regain ambulation. Here, we describe the case of a mid-octogenarian who presented with severe pain and nonambulatory quadriparesis attributable to MESCC at the fifth cervical vertebra, which developed 10 months after the diagnosis of undifferentiated carcinoma of the gallbladder. Image-guided three-dimensional conformal radiotherapy (IG-3DCRT) was started with 25 Gy in 5 fractions followed by a boost of 12 Gy in 3 fractions, for which a field-in-field (FIF) technique was used to optimize the dose distribution. Despite the fact that steroids were not administered, the patient reported significant pain reduction and showed improved motor function 3 and 4 weeks after the IG-3DCRT, respectively. Over the following 4 months, her neurological function gradually improved, and she was consequently able to eat and change clothes without assistance and to walk slowly for 10–20 m using a walker. She succumbed to progression of abdominal disease 8.5 months after the IG-3DCRT. This case demonstrates that image-guided FIF radiotherapy with a dose-escalated hypofractionated regimen can potentially improve functional outcome and local control.

## 1. Introduction

Metastatic epidural spinal cord compression (MESCC) is a common neurological complication of cancer and can have devastating consequences for patients [1–3]. Radiotherapy, either alone or following surgical decompression, still has a pivotal role in the management of MESCC [1–3]. Several factors affect functional outcome, the most important of which is the level of preserved neurological function before treatment (i.e., ambulatory status) [1–3]. Patients who are paraplegic before radiotherapy rarely regain ambulation, and this in turn can lead to a number of health problems related to immobility [2]. Furthermore, elderly patients, especially octogenarians, might be more susceptible to neurological damage by MESCC and their health is also likely deemed as poor to allow them to recover.

Despite the growing sophistication of radiotherapy techniques such as intensity-modulated radiotherapy (IMRT) and stereotactic body radiotherapy (SBRT) [2, 3], conventional methods that use opposed 2 ports are still commonly used to treat acute symptomatic MESCC, probably because treatment must be initiated immediately [1, 3]. However, image-guidance systems allow conventional radiotherapy to be delivered more precisely with the use of a reduced setup margin [4]. Furthermore, a field-in-field (FIF) technique has been applied to three-dimensional conformal radiotherapy (3DCRT) for various indications to amend dose distribution [5, 6]. However, to our knowledge, the use of this technique to treat spinal metastases, including MESCC, has not previously been reported.

Here, we describe the case of a mid-octogenarian who presented with severe pain and nonambulatory tetraparesis attributable to acute MESCC from gallbladder carcinoma. This patient was treated with image-guided 3DCRT (IG-3DCRT) alone using a FIF technique and a unique hypofractionated dose fractionation scheme, which resulted in good tumor control and sufficient cord decompression, which in turn allowed a delayed but gradual improvement in her severe neurological dysfunction.

## 2. Case Report

An 85-year-old woman presented with epigastric discomfort and was clinically diagnosed with locally advanced unresectable carcinoma of the gallbladder associated with liver metastases. The histopathological diagnosis was undifferentiated carcinoma. The patient initially underwent 4 courses of systemic chemotherapy that consisted of cisplatin and gemcitabine, resulting in a partial response, but the disease subsequently progressed. Transarterial chemoembolization (TACE) was then administered 3 times using epirubicin, mitomycin C, and 5-fluorouracil along with degradable starch microspheres, and this again resulted in a partial response. Thereafter, the patient received 80 mg/day of S-1 (tegafur, 5-chloro-2,4-dihydropyrimidine and potassium oxonate) with 1 week of rest after 3 weeks of administration as a maintenance treatment.

**Figure 1:**
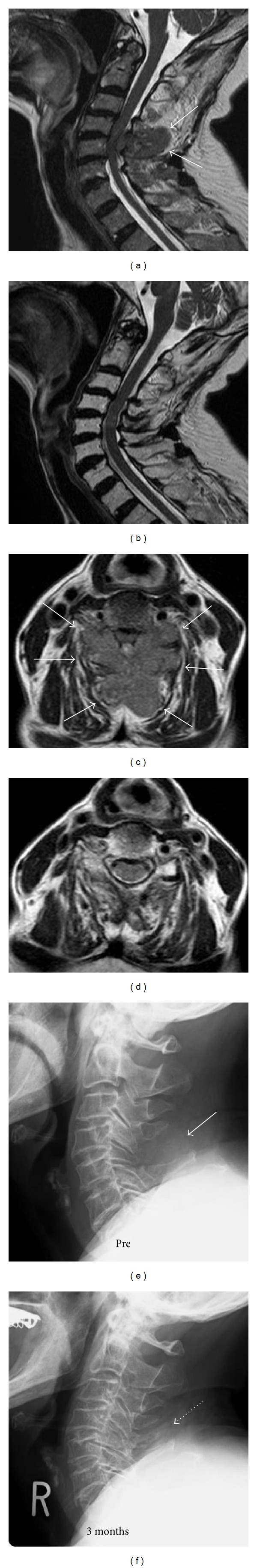
Magnetic resonance images: (a), (b) sagittal views; (c), (d) axial view at the fifth cervical vertebra (C5); and (e), (f) plain radiographs before (a), (c), and (e) and 3 months after (b), (d), and (f) radiotherapy. The arrows (a), (c) indicate the epidural mass involving the spinous process, laminae, and pedicles at C5 and compressing the spinal cord circumferentially. Plain radiographs showing an osteolytic change in the spinous process at C5 (arrow) and subsequent reossification (dashed arrow).

Although the primary cancer and liver metastases remained stable thereafter, the patient subsequently presented with severe neck/shoulder pain, difficulty in walking, clumsy hands, and numbness in both upper extremities 10 months after the initial diagnosis. Imaging findings revealed malignant epidural compression at the fifth cervical vertebra (Figures 1(a), 1(c), and 1(e)). The lesion was located predominantly in the posterior element and compressed the spinal cord circumferentially (Figure 1(c)), while the physiological alignment of the cervical spine was almost preserved without any instability (Figures 1(a) and 1(e)). The patient's neck was immediately immobilized with a collar, but the pain was refractory to analgesics selected according to the WHO analgesic ladder. The patient's neurological symptoms relentlessly worsened to severe tetraparesis during imaging surveillance and while the orthopedicians were considering the best treatment strategy, emergent surgical decompression was initially considered, but the patient and her family declined it. Accordingly, the patient was referred to us for radiotherapy 6 days after the visit, but at this time, she was already nonambulatory and could not maintain a sitting position or even adopt the semi-Fowler's position, owing to severe pain and quadriparesis. The only perceived motor function was slight movement in the right elbow joint. After obtaining informed consent, radiotherapy was initiated the following day, 18 hours after the referral. We also recommended the immediate administration of steroids, but an attending practitioner declined to use these.

**Figure 2:**
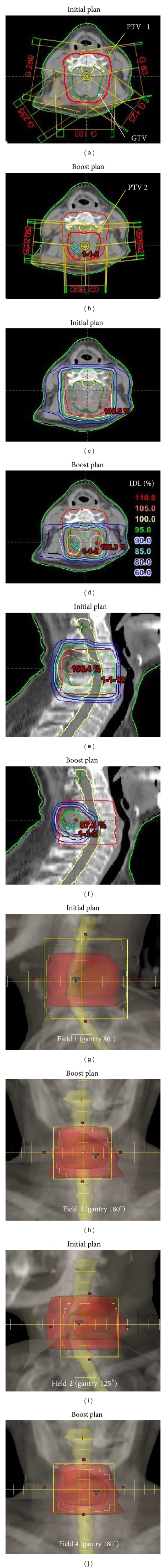
Target definitions, beam arrangements, and dose distributions for initial (left) and boost (right) plans. Beam arrangements (a), (b); dose distributions ((c), (d): axial; (e), (f): sagittal views); and beam's eye views (g)–(j) for some representative fields.* IDS* isodose line.

IG-3DCRT was delivered using a Clinac iX (Varian Medical Systems, Inc., Palo Alto, CA, USA) with a central leaf width of 5 mm and 6-MV X-rays, commissioned with the Eclipse ver 8.6 (Varian), which was equipped with an on-board imager (OBI) (Varian). Patients were immobilized with a general thermoplastic mask. Planning computed tomography (CT) images were acquired with a 3 mm slice thickness. The gross tumor volume (GTV, 35.9 cm^3^) was contoured on the CT images with reference to magnetic resonance images, and the planning target volume (PTV, 168.9 cm^3^) consisted of the fourth to sixth cervical vertebrae, including the GTV with an additional 3 mm isotropic setup margin. Dose calculations were based on a pencil beam convolution in conjunction with the Batho power law for tissue heterogeneity correction. The treatment plan was then generated using 5 static coplanar multibeams with different weights, by adjusting the leaf margins anisotropically to ensure that the PTV and GTV were encompassed by 95% and 100% of the isodose surfaces, respectively, and the highest dose was located within the GTV (Figures 2(a), 2(c), 2(e), 2(g), and 2(i)). The prescribed dose was 25 Gy in 5 fractions to the reference point near the PTV isocenter. Image-guided radiotherapy was implemented via online 3D correction based on the results of the 2D-2D matching. Although the planned 5-fraction radiotherapy could be administered, no obvious improvement in neurological function was observed during this treatment. Subsequently, a boosting dose of 12 Gy in 3 fractions focusing on the GTV was planned, with particular emphasis on the cumulative dose to the affected cord, and this was administered sequentially after obtaining additional informed consent. The PTV (42.0 cm^3^) for the boost consisted of the GTV without the dural theca and setup margin. The FIF technique was used to optimize the dose distribution, thereby ensuring that the dural theca was spared, with a dose <90% of the isodose surface (Figures 2(d), 2(f), 2(h), and 2(j)). The prescribed dose was specified to the reference point located in the right side of the GTV (Figure 2(d)), and the planned treatment was completed over a period of 10 days. The only notable acute adverse reaction was grade 2 dysphagia attributable to pharyngeal mucositis, but this subsequently resolved. The details of the beam arrangements and dosimetric data are given in Table 1.

The posttreatment clinical course is summarized in Table 2. No obvious neurological improvement was observed for more than 2 weeks after the completion of radiotherapy, although there was significant pain reduction after 10 days. The patient continued to undergo bedside rehabilitation to mitigate disuse syndrome. One month after the initiation of radiotherapy, the patient's motor dysfunction began to improve gradually, first in the right upper extremity, then in the left upper extremity, and finally in the lower extremities (Table 2). Despite the fact that no bone strengthening agents were administered, the follow-up images obtained 3 months after radiotherapy revealed a favorable tumor response, adequate decompression of the affected cord without abnormal parenchymal intensity, and preservation of physiological spinal alignment along with reossification (remodeling) of the posterior element of the C5 vertebra, although bulging of the ligamenta flava was still observed (Figures 1(b), 1(d), and 1(f)). Eighteen weeks after the IG-3DCRT, asymptomatic progression of the primary cancer was evident, for which TACE was attempted, but this did not allow a sufficient dose to be administered. The patient was transferred to another hospital to continue rehabilitation 29 weeks after radiotherapy. At that time, she was able to eat with a fork and change her clothes without assistance. Her grip strengths were 5.0 and 1.5 kg in the right and left hands, respectively, and she was able to walk 10–20 m with the aid of a walker. She had minimal numbness in the upper extremities and required no further analgesia. She had only a modest urge to evacuate her bowels and had occasional urinary/fecal incontinence. One month later, her general condition worsened owing to progression of the primary cancer and liver metastases, resulting in her death 8.5 months after the IG-3DCRT (19 months after the initial treatment). During the clinical course, there were no obvious findings suggestive of L'Hermitte's sign antecedent to myelopathy.

## 3. Discussion

Despite rapid neurological aggravation leading to the loss of ambulation and almost complete quadriplegia before the start of radiotherapy, a delayed but gradual improvement of neurological function, leading to a partial recovery with limited ambulation, was achieved for this mid-octogenarian patient. In addition, follow-up images showed a favorable tumor response associated with sufficient decompression of the affected cord and preservation of the physiological alignment of the affected spine along with bone remodeling.

This case differed from others involving MESCC patients treated with radiotherapy with respect to (1) gallbladder carcinoma being the cause of MESCC, (2) the patient being a mid-octogenarian, (3) the use of IG-3DCRT with a FIF technique for radiotherapy, (4) the use of a 2-step approach for target definition and dose fractionation, and (5) dose escalation using a hypofractionated regimen. In addition, there were several disadvantageous factors affecting the clinical outcome, including the patient's age, severe neurological dysfunction before radiotherapy, and nonadministration of steroids or bone strengthening agents.

Gallbladder carcinoma is a rare cause of MESCC [7]. The median survival time for patients with advanced gallbladder carcinoma treated with cisplatin/gemcitabine is almost 12 months [8], while the overall survival in the present case was 19 months. Radiotherapy remains an integral part of the treatment for gallbladder carcinoma, especially in cases not amenable to curative resection, while adenocarcinoma of the gallbladder (the more common histological type) is generally deemed to be less sensitive to conventional radiotherapy.

Various dose fractionation regimens have been so far investigated and used for MESCC, including 8 Gy in one fraction, 12 or 16 Gy in two fractions, 20 or 25 Gy in 5 fractions, 24 Gy in 6 fractions, 15 Gy in 3 fractions followed by 15 Gy in 5 fractions, 30 Gy in 10 fractions, 35 Gy in 14 fractions, 37.5 Gy in 15 fractions, and 40 Gy in 20 fractions [9–16]. However, few paraplegic patients regain ambulation following these conventional radiotherapy regimens irrespective of a number of fractions [2, 10, 12, 13, 15]. We have previously also used standard dose fractionation schemes for acute MESCC, such as 30 Gy in 10 fractions, 20 Gy in 5 fractions, or 8 Gy in a single fraction with dose escalation for selected cases, especially when image guidance was unavailable. However, in our experience, these schemes have never provided a significant improvement in paraplegia from MESCC. In the case we report here, we instead used dose escalation, especially to the GTV, using a hypofractionated scheme via a FIF technique under image guidance. The median cumulative dose to the GTV was 45.3 Gy administered as a 2 Gy/fraction equivalent dose (Table 1(b)). Furthermore, the inhomogeneous dose distribution within the PTV was intended to increase the dose to the GTV while reducing the dose to the spinal cord and posterior pharyngeal wall through adjusting the leaf margin and beam weight, in addition to the use of the FIF method. Conventional radiotherapy techniques such as opposed two ports or posterior one port do not necessarily provide suitable dose distribution with consideration of the relationship between the tumor burden and the affected cord. Thus, the dose fractionation scheme and the dose distribution presented here were completely different from those previously used.

The risk of myelopathy after conventional radiotherapy is estimated as <10% for a 2 Gy/fraction equivalent dose of 61 Gy to the cervical cord [17], while the cumulative maximum dose to the affected cord in this case was 70.4 and 60.8 Gy administered as a 2 Gy/fraction equivalent dose with *α*/*β* values of 0.87 and 2, respectively (Table 1(b)) [17]. The cervical cord was expediently contoured as the dural theca [18] and the contouring was rather overdrawn. The actual dose delivered to the affected cord can also be influenced by the daily accuracy of setup and immobilization and the potential early tumor response during the course of radiotherapy. Moreover, dose distribution to the affected cord in this case was substantially heterogeneous (Table 1(b)), while the tolerance dose to the spinal cord was largely extrapolated from either conventional fractionation to the almost whole cord or extreme hypofractionation to part of the cord [17, 18]. Nonetheless, we could not draw any conclusion as to the appropriateness of the delivered dose to the affected cord given the limited observation period, and it deserves further investigation.

Taken together, the delayed but gradual improvement of neurological dysfunction in this case was probably attributable to (1) the favorable radiosensitivity of the epidural tumor, the histological analysis of which suggested that it was derived from an undifferentiated tumor type; (2) the preservation of the physiological alignment without instability, which was possible because the tumor was primarily located in the posterior elements of the affected spine; and (3) the relatively high and precisely delivered dose to the tumor with a reduced dose to the affected cord. The latter was achieved using image guidance and the FIF technique, although the pathology of the epidural lesion was not ascertained.

If an adequate steroid dose had been administered immediately after the diagnosis of MESCC and the adverse reactions had been appropriately managed, the functional outcome might have been better in this case. Furthermore, given the possibility of delayed and indolent functional recovery in cases of paraplegia resulting from MESCC, the continuation of appropriate rehabilitation would be important to prevent progression of disuse syndrome without hindering functional recovery in the early stages.

If treatment needs to be initiated quickly and short irradiation time is required in cases similar to this, in which it is difficult to keep the patient in the treatment position for a prolonged period, it may be preferable to initially use a conventional technique and subsequently replace this with 3DCRT using a FIF technique when feasible. To shorten treatment time in such cases, it might be better to change the gantry angles of the low-weight beams in the initial plan from 125° and 235° to 80° and 280°, respectively, making it similar to the FIF approach.

Both the dose fractionation scheme and FIF planning still need to be improved for cases such as the one we report here. Nevertheless, our experience in this case suggests that IG-3DCRT using a FIF technique and a dose-escalated hypofractionation regimen can potentially provide better clinical outcomes than conventional schemes. Given the limitations of advanced but complicated and expensive radiotherapy techniques such as IMRT and SBRT in these oncological emergencies, the simple modification of conventional radiotherapy techniques would seem to be both practical and imperative. Furthermore, the treatment cost for the presented IG-3DCRT with 8 fractions was deemed comparable to those for conventional radiotherapy, given that IGRT is recently applied to palliative radiotherapy for most patients harboring spinal metastases, and treatment cost basically also depends on a number of dose fractions. In this regard, a clinical study of IG-3DCRT using a FIF method along with further optimization of dose fractionation schemes is justified to improve the functional and oncological outcomes of MESCC patients.

## Figures and Tables

**(a):** 

	Field (number)	Gantry rotation (°)	Collimator angle (°)	Weight	Illustrations
Initial plan	1	80	0	1.00	Figure 2(g)
	2	125	0	0.07	Figure 2(i)
	3	180	0	0.80	
	4	235	0	0.07	
	5	280	0	1.00	

Boost plan	1	85	0	0.95	
	2	85	0	0.07	
	3	180	0	0.30	Figure 2(h)
	4	180	90	0.18	Figure 2(j)
	5	275	0	1.00	
	6	275	0	0.05	

**(b):** 

Spinal cord dose^a^	*D*_max⁡_	*D*_0.1 cc_	*D*_1 cc_	*D*_2 cc_	*D*_5 cc_	*D*_10 cc_
Initial plan (%)	101.6	101.0	100.4	100.0	98.0	77.5
Boost plan (%)	92.6	90.0	82.8	71.5	30.0	4.0
Cumulative BED_2_ (Gy)	121.6	119.2	114.5	108.3	90.3	57.4
Cumulative BED_0.87_ (Gy)	232.1	227.3	217.7	205.5	171.1	106.2
2 Gy dose equivalent (*α*/*β* = 2) (Gy)	60.8	59.6	57.2	54.2	45.1	28.7
2 Gy dose equivalent (*α*/*β* = 0.87) (Gy)	70.4	68.9	66.0	62.3	51.9	32.2

GTV dose	*D*_98_	*D*_95_	*D*_90_	*D*_50_	*D*_mean_	*D*_2_

Initial plan (%)	98.2	98.8	102.0	102.0	102.3	106.0
Boost plan (%)	44.0	59.0	95.8	95.8	91.5	101.8
Cumulative BED_10_ (Gy)	42.8	45.7	54.4	54.4	53.7	57.7
2 Gy dose equivalent (*α*/*β* = 10) (Gy)	35.7	38.0	40.8	45.3	44.7	48.1

PTV dose	*D*_98_	*D*_95_	*D*_90_	*D*_50_	*D*_mean_	*D*_2_

Initial plan (%) [PTV 168.9 cm^3^]	90.0	93.0	94.4	99.0	99.2	105.8
Boost plan (%) [PTV 42.0 cm^3^]	86.0	89.7	91.8	97.5	96.8	102.0

*D*_max⁡_: maximum dose, BED: biological effective dose, *D*_mean_: mean dose, GTV: gross tumor volume, and PTV: planning target volume. *D*_*n*_ (%) represents the dose (%), relative to the prescribed point (100%), receiving at least *n* % of volume and *D*_*n* cc_ (%) means the dose (%) receiving at least *n* cm^3^ of volume. BED is calculated according to the linear-quadratic formula: BED_*n*_ (Gy) = total dose × [1 + (dose per fraction)/*n*], where *n* represents the *α*/*β* ratio (*α*/*β* = 10 for the antitumor effect; *α*/*β* = 2 and 0.87 for spinal cord), and 2 Gy dose equivalent (*α*/*β* = n) is the 2 Gy per fraction equivalent total dose according to the *α*/*β* value of *n*. ^a^The spinal cord is expediently contoured as the dural theca.

**Table 2:** Clinical course after the commencement of radiotherapy.

Time course	Neurological function and other events
Days 1–10	Radiotherapy (days 5, 6: none)
Day 20	Significant pain reduction
Day 23	Being able to keep the Fowler's position
**Day 29**	Improvement in the right elbow movement
Day 38	Being able to keep sitting under full assistance
Day 39	Being able to move the right fingers (pinching)
**Day 45**	Being able to move the **left** upper extremity/removal of urethral catheter
Day 49	Being able to keep sitting without assistance
**Day 50**	Being able to move the **lower extremities**
Day 56	Significant decrease in numbness of the upper extremities
**Day 65**	Being able to **keep standing** under holding onto parallel bars
Day 77	Being able to perform stepping in place
**Day 85**	Initiation of **gait training **in parallel bars
**Day 120**	Being able to **walk slowly** with the aid of a walker
Day 128	Attempt at transarterial chemoembolization
Day 203	Transfer to another hospital for continuing rehabilitation
Day 238	General condition declined owing to progression of abdominal disease
**Day 258**	Deceased
